# Breast assessment using next generation handheld ultrasound device based on silicon chips: a pilot study in senology

**DOI:** 10.1007/s00404-026-08326-1

**Published:** 2026-01-24

**Authors:** R. Plöger, C. Ludwig, G. Nowozin, K. Winkler, A. Abramian, A. Faridi, F. Recker

**Affiliations:** 1 Department of Obstetrics and Prenatal Medicine, University Hospital Bonn, Venusberg Campus 1, 53127 Bonn, Germany; 2 Department of Senology, GYNCOLLEGWESERLAND Eidinghausen, Eidingsen 2, 32549 Bad Oeynhausen, Germany; 3 Department of Senology, University Hospital Bonn, Venusberg Campus 1, 53127 Bonn, Germany; 4 Department of Obstetrics, University of Cologne, Faculty of Medicine and University Hospital Cologne, Kerpener Straße 34, 50931 Cologne, Germany

**Keywords:** Portable ultrasound, Point-of-care ultrasound, Handheld devices, Breast cancer, Breast fibroadenoma, Diagnostic accuracy, Outpatient monitoring, Semiconductors, Technology, Piezoelectricity

## Abstract

**Background:**

In breast care, ultrasound examination is a very important tool used to detect breast tumors, to monitor core needle biopsies, for preparing surgical operations, and for tracking postoperative developments. So far, stationary high-end ultrasound devices (SHUD) based on piezoelectric technology are most commonly used but lack the mobility and thus the possibility to practice point-of-care ultrasound (POCUS) in senology. In contrast, handheld ultrasound devices based on silicon chips (HHUD) offer a high mobility and different penetration depths through its all-in-one probe principle and thus may improve patient treatment. Therefore, this study investigates the diagnostic reliability of breast lesions examined with HHUD based on silicon chips versus those examined with SHUD based on conventional piezoelectric technology.

**Methods:**

Each patient received an ultrasound examination using SHUD (Voluson S10, GE Healthcare) and HHUD (Butterfly iQ, Butterfly Network) in a random order. The morphologic descriptors and the BI-RADS categories, as well as the histological results, in the case of the biopsy, were compared, and the agreement rate and the Cohen’s kappa were analyzed. A quantitative analysis of the lesions’ sizes examined by the two devices was assessed statistically through intra-class correlation coefficient (ICC), Bland–Altman plots, and Pearson correlation coefficient (PCC). Subgroup analysis was performed in lesions’ type, skin-to-lesion distance, and lesions’ volume.

**Results:**

105 lesions found in 84 females were analyzed regarding the reliability of SHUD and HHUD. The BI-RADS categories matched perfectly between both the devices and the available histological outcomes. The agreements of the measured diameters were excellent (ICC 0.926–0.969). The subgroup analysis revealed a slightly superior agreement for malignant cases, for lesions over 0.5 ml and for a skin-to-lesion distance over 5 mm.

**Conclusion:**

The categories and measurements from HHUD matched closely with those obtained using conventional SHUD. This research demonstrates that HHUD offers a good alternative to SHUD for breast lesion evaluation which becomes especially useful during point-of-care applications.

**Supplementary Information:**

The online version contains supplementary material available at 10.1007/s00404-026-08326-1.

## Background

Ultrasound technology is essential in obstetrics and gynecology as seen by the multiple applications including the examination of reproductive organs, fetal development, and breast tissue [1]. In breast health, ultrasound devices are used for preventive examinations of the breast tissue, for the evaluation of lesions, for monitoring during needle biopsies and during breast surgery, as well as for postoperative control examinations [2]. Sonographic findings can be classified using the breast imaging reporting and data system (BI-RADS) allowing risk assessment and clinical management decisions [3]. The need for ultrasound technology in senology for detecting and handling breast diseases has become evident through extensive documentation over the last decades [2], whereby stationary high-end ultrasound devices (SHUD) based on piezoelectric technology are most commonly used. However, this clinical practice of using SHUD faces several limitations in patient care. In the case of postoperative complications, the patients must be transported into certain examination rooms equipped with a SHUD, thereby delaying the sonographic diagnosis. Intraoperatively, SHUD are known to improve the rate of tumor-free margins in palpable and non-palpable sonographically visible breast tumors [4, 5] but are often not available in operating theaters. Preoperatively, the installation of SHUD in specific facilities provides severe barriers for women unable to visit these centers [6–8]. Thus, the application of transportable ultrasound devices provides benefits for physicians and patients in all these exemplary settings.

Handheld ultrasound devices may be a logical choice in these scenarios. These ultrasound devices consist of a probe which connects to smartphones or tablets, making them easily transportable. The compact handheld ultrasound devices fit inside a lab coat pocket like stethoscopes [9] and thus enable a quick and location independent use already seen in other specialties, such as emergency and internal medicine [10–12]. Nowadays, almost every ultrasound manufacturer produces their own handheld ultrasound device [13, 14], which differ in their features. One of these handheld ultrasound devices uses a new ultrasound technology based on silicon chips (HHUD) [15, 16]. This new technology enables various clinical applications due to the different possible ultrasound frequencies of one probe, an “all probes in one” principle. The key component of these probes is capacitive micro-machined ultrasound transducers: using electrical voltage, a capacitive membrane starts to vibrate and thus generates ultrasound waves whose frequency depends on the voltage [15, 16]. In contrast, the piezo material in the conventional probes defines a specific ultrasound frequency so that several probes are needed to perform the entire spectrum of obstetric and gynecologic examinations. Using one and the same probe, the HHUD can be used to localize Implanon^®^ subcutaneously using a high-frequency setting [17] and also be used to examine fetal biometry [18–20] or postpartum complications [21, 22] using a low-frequency setting. The correlation measured by Pearson correlation coefficient (PCC) and agreement measured by the intra-class correlation coefficients (ICC) between HHUD and SHUD was near perfect for the measured parameters [18–22]. Thus, an application in breast care could be possible too. The research literature regarding handheld ultrasound devices as diagnostic tools for breast disease evaluation is not extensive despite their proven advantages in other gynecological and obstetric specializations [17–22]. This is particularly notable giving the global incidence of breast diseases, especially breast cancer [23]. Interestingly, subgroup analysis of HHUD in obstetrics revealed that the agreement and correlation of uterine horizontal axis measurements between HHUD and SHUD are lower in patients with normal weight than in overweight patients. One explanation could be the reduced expansion of the acoustic window due to the lack of subcutaneous tissue [21]. However, this observation needs confirmation in studies with higher numbers and in other contexts. HHUD seems to be easily integrated into clinical work flow: ultrasound skills and knowledge can be transferred from SHUD to HHUD [24] and may be the new standard ultrasound device for the upcoming generation of physicians due to the increasing implementation of HHUD in medical curricula and residency programs [25–27].

Based on these observations, the handheld ultrasound devices based on silicon chips may be applied in the diagnostics and treatment of breast diseases. This could revolutionize the use of ultrasound devices for the next generation of physicians who train with handheld devices from early on and regularly work in mixed wards with senologic, obstetric, and gynecologic patients. The HHUD allow an examination in these different application areas but must prove its reliability in senology. Thus, the aim of this study is to analyze whether handheld ultrasound devices based on silicon chips using a high-frequency setting can be used for breast ultrasound examinations.

## Materials and methods

### Study design

This study was designed as a prospective, comparative analysis to evaluate the diagnostic reliability of HHUD in comparison to SHUD in the management of breast lesions in two breast centers (University Hospital Bonn, Bonn, Germany and GYNCOLLEGWESERLAND Eidingsen, Bad Oeynhausen, Germany), which are highly specialized in the diagnosis and treatment of breast diseases. Informed consent was obtained from all participants. The research protocol was received and approved by the ethics committee of the University Hospital Bonn (reference number 368/23-EP). All breast ultrasound examinations followed the examination guidelines of the German society for ultrasound (DEGUM) [28] and international standards [3].

### Ultrasound devices

**Fig. 1:**
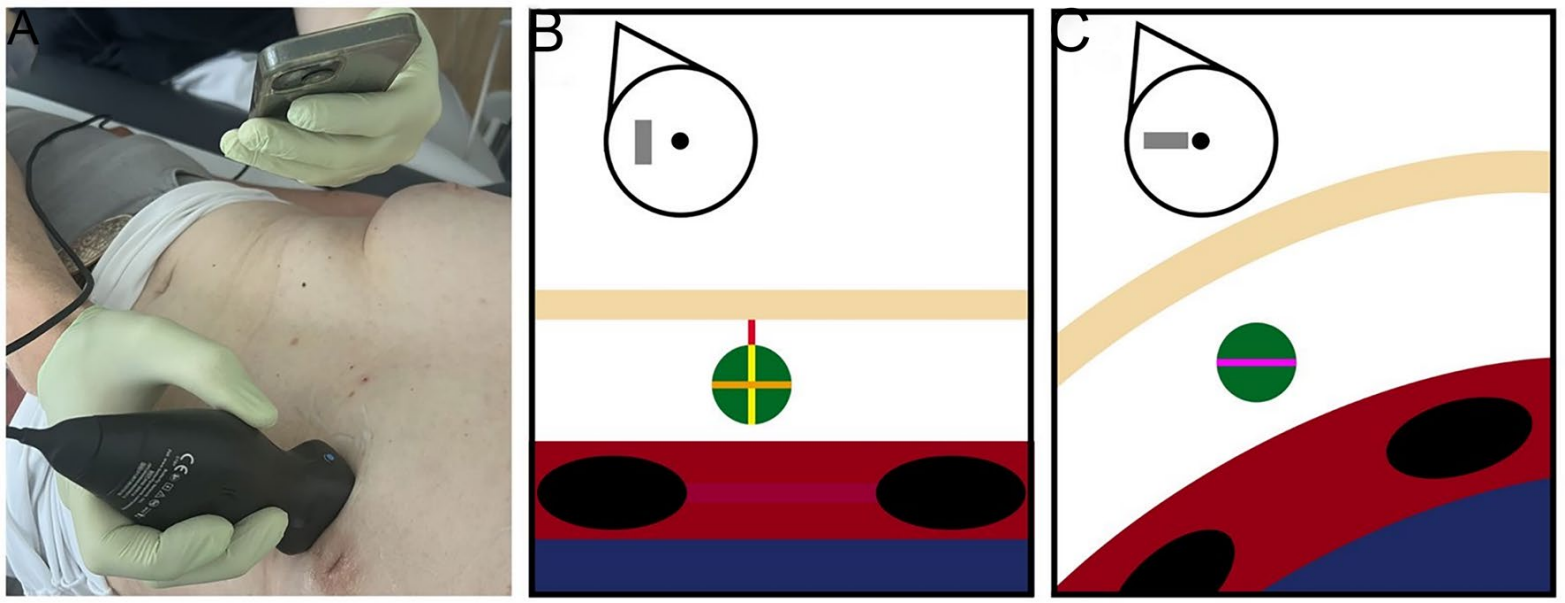
Clinical setting during a breast examination using handheld ultrasound device (**A**) and schematic modeling of the lesions during the ultrasound breast examinations : A patient’s examination using a handheld ultrasound probe for lesion localization (**A**). Schematic modeling of a lesion in sagittal (**B**) and vertical planes (**C**). The transducer orientation marker is shown in the upper right corner. The ultrasound waves pass through the skin (beige band), then the breast tissue containing the lesion (green circle), and finally reach the underlying muscle layer (red) with rib shadows (black ovals) and lungs (blue). Craniocaudal diameter (orange), dorsoventral diameter (orange) and mediolateral diameter (purple) and skin-to-lesion distance (red)

This study used two different ultrasound devices with two different technological approaches: (1) The SHUD, in this case, the Voluson S10 from General Electric using the linear transducer ML6-15, is a stationary ultrasound device based on piezoelectric technology and is commonly used in clinical settings for detailed obstetric and gynecological imaging. This system uses piezoelectric crystal transducers that convert electrical signals into mechanical vibrations to produce ultrasound waves. These waves are reflected back to the transducer, allowing the formation of high-resolution images. The SHUD’s probe used in the study offers a high frequency, providing the resolution and depth penetration necessary for detailed breast imaging and is the reference standard in this context. (2) The HHUD, represented by the Butterfly iQ, uses a silicon chip-based transducer. This device employs semiconductor technology to generate ultrasound waves with several settings: based on the settings, different possible frequencies of the ultrasound waves can be generated by one probe leading to different penetration depth and resolution (“all probes in one” principle). Furthermore, the user can switch between linear and convex modes. The HHUD’s setting was a linear transducer with a frequency range comparable to the SHUD allowing for similar imaging capabilities. However, the HHUD’s design emphasizes portability as it can be connected to a smartphone or tablet, facilitating point-of-care examinations and analysis on mobile platforms (Fig. 1).

### Examination protocols

 Patients were informed about the study following diagnosis of a breast lesion using a conventional ultrasound device. Only patients with a certain breast density, allowing an examination using ultrasound, were included. Patients with high breast density, matching category D [3, 29], as well as patients with disabilities were excluded. Once informed consent was obtained, the examiners, who lacked knowledge about the lesion, repeated the examination using both devices, SHUD and HHUD, in a random order. The examinations were conducted in a controlled environment to minimize external variables that could affect image quality or diagnostic outcomes.

**Fig. 2:**
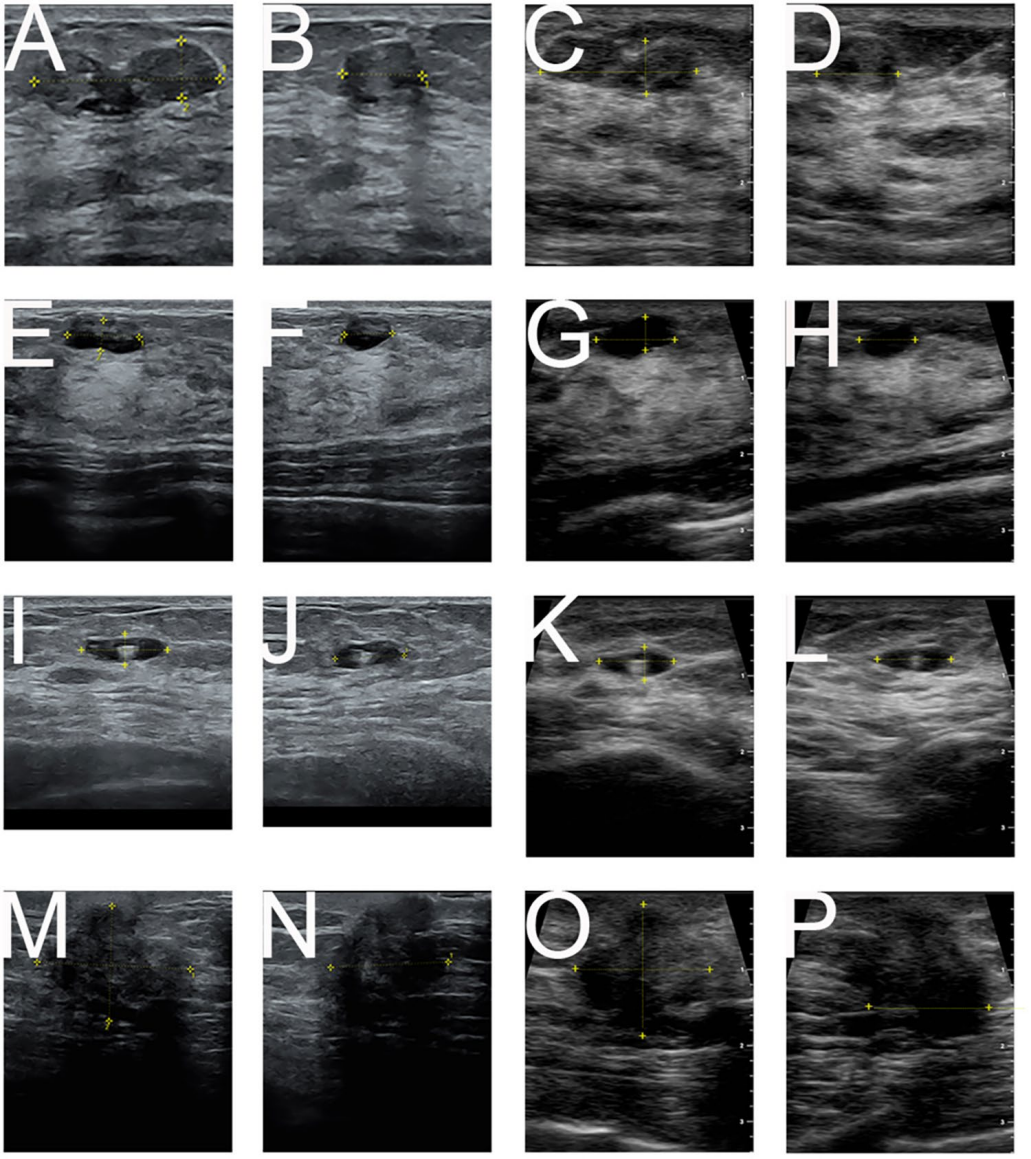
Ultrasound images of breast lesions: fibroadenoma (**A**–**D**), cyst (**E**–**H**), lymph node (**I**–**L**) and carcinoma (**M**–**P**) examined using a stationary high-end  ultrasound device (SHUD, A-B, E–F, I-J, M–N) and a handheld ultrasound device (HHUD, C-D, G-H, K-L, O-P)

After the examination was completed and images were saved, the morphologic descriptors were carefully assessed using both devices. The lesions were characterized using the established morphological descriptors and classified according to the BI-RADS categories [3, 28]. No abnormalities found were labeled as BI-RADS category 1. Benign lesions such as cysts or stable scars were labeled as BI-RADS category 2 and needed routine screening. Probably benign lesions with a likelihood of cancer under 2% were categorized as BI-RADS category 3. Suspicious lesions labeled as BI-RADS category 4 and highly suspicious lesions labeled as BI-RADS category 5 required a biopsy. Based on the BI-RADS categories, histological examinations were performed. According to medical guidelines, no biopsies were performed on lesions in BI-RADS categories 1–3 [3, 28, 29]. For each lesion, three diameters and the skin-to-lesion distance were precisely measured and documented. The volumes of the lesions (in ml) were measured using the ellipsoid volume formula suiting for the shape of the lesions: π/6 × craniocaudal diameter x dorsoventral diameter x mediolateral diameter. All examinations were performed by experienced sonographers certified as DEGUM level I breast ultrasound examiners (RP, KW and AA) (Fig. 2).

### Statistical analysis

Statistical analysis and visual data presentations were performed using the statistical package for the social sciences (SPSS) software, version 27 (IBM Corp., Armonk, NY, USA). The sample size of 100 breast lesions without lymph nodes was selected based on the size of similar pilot studies [18, 20–22], the practical recruitment considerations, and the likelihood to enclose a broad spectrum of lesions. The agreement rate and Cohen's kappa between the morphologic descriptors and the BI-RADS categories based on the examination with a SHUD and HHUD were calculated. A Cohen’s kappa under 0 indicates a poor strength of agreement, between 0 and 0.2 indicates a slight strength of agreement, 0.21 and 0.4 indicates a fair strength of agreement, 0.41 and 0.6 indicates a moderate strength of agreement, 0.61 and 0.8 indicates a substantial strength of agreement, and 0.81 and 1.00 indicates an almost perfect strength of agreement [30]. To assess the reliability and consistency of measurements obtained with SHUD and HHUD, ICC employing a two-way random effect, agreement model with a 95% confidence interval (95% CI) and Bland–Altman plots were used. In the case of a low ICC and thus low absolute agreement between the examinations using HHUD and SHUD, the PCC would help distinguish between a systematic bias if a high PCC and a low ICC were seen, or a random error if a low PCC and a low ICC were seen [31]. Therefore, PCC were calculated to be prepared for a further analysis—in the case of low ICC—and to allow a comparison with other studies displaying the PCC in the context of reliability between HHUD and SHUD [18, 20–22, 24]. An ICC and a PCC approaching the value of 1.0 indicate a high degree of agreement (assessed by ICC) or correlation (assessed by PCC) [32–34]. In the case of ICC, values less than 0.5 indicate poor agreement, between 0.5 and 0.75 moderate agreement, between 0.75 and 0.9 good, and values greater than 0.9 excellent agreement [34]. Bland–Altman plots [35] were utilized as a graphical method to assess agreement by plotting the differences between measurements against their averages, helping to visualize potential systematic bias or variability between the two methods. These combined statistical approaches allowed for a comprehensive evaluation of the performance and agreement between SHUD and HHUD. Subgroup analysis was performed regarding lesions’ type, skin-to-lesion distance and lesions’ volume. The criteria for skin-to-lesion distance and lesions' volume were under or over 5 mm or 0.5 ml, respectively, so that both groups have similar numbers.

## Results

This prospective study analyzed 105 breast lesions from 84 female patients. The participants’ age ranged from 17 to 84 years. The median age was 51.5 years. The BMI values ranged from 18.4 to 41.8 kg/m^2^, with a median BMI of 23.7 kg/m^2^. The patients with available BMI data showed that 32 patients (41.6%) exceeded the BMI threshold of 25 kg/m^2^, defining  overweight.

Sonographic criteria for breast lesions using BI-RADS criteria could be described as similar independent of the use of a HHUD or SHUD (Table 1). The agreement of the morphologic descriptors and the BI-RADS categories obtained by HHUD or SHUD are very high. The Cohen’s Kappa shows an almost perfect strength of agreement [30]. Based on the BI-RADS categories, 66 histological examinations were performed, and ultimately the histological results matched the previously determined BI-RADS categories. (Table 2). The mamma carcinomas formed the largest subgroup with 37 cases followed by 32 cysts and 22 fibroadenomas (Table 2). In 39 cases, the lesions were assumed benign based on imaging (BI-RADS 1–3) and not histologically verified.

**Table 1:** Cohen’s kappa and agreement rate of the morphologic descriptors and BI-RADS categories using a stationary high-end ultrasound device (SHUD) and a handheld ultrasound device (HHUD) for the 105 lesions

Morphologic descriptors	SHUD	HHUD	Cohen’s kappa	Agreement rate
**Shape**			1	100%
oval	32	32		
round	33	33		
irregular	40	40		
**Orientation**			1	100%
parallel	63	63		
non-parallel	42	42		
**Margin**			1	100%
distinct	65	65		
indistinct	33	33		
spiculated	7	7		
**Echo pattern**			1	100%
hypoechoic	95	95		
hyperechoic	10	10		
**Posterior features**			1	100%
enhancement	38	38		
shadowing	67	67		
**BI-RADS Categories**			1	100%
BI-RADS 0	0	0		
BI-RADS 1	0	0		
BI-RADS 2	38	38		
BI-RADS 3	1	1		
BI-RADS 4	26	26		
BI-RADS 5	40	40		
BI-RADS 6	0	0

**Table 2:** Lesions with their respective  number of histological examinations (HE, in number), with their relative rate of histological examinations (HER, in percent), with their agreement rate between histological examination and ultrasound examination (AR, in percent), their volume and their skin-to-lesion distance (*n* = 105) categorized as benign (BEN) or malignant (MAL) lesions. The ductal carcinoma in situ (DCIS) is categorized as a malignant lesion. 63% of the sonographic diagnoses were histologically controlled. 39 lesions were not histologically analyzed due to their benign appearance in the ultrasound. The volume of the lesions were calculated using the following formula: π/6 × craniocaudal diameter x dorsoventral diameter x mediolateral diameter. The range of the volume, median and mean volume are stated in ml. The unit for the skin-to-lesion distance is mm. Lymphnode (node)

		HE	HER	AR	Volume			Skin-to-lesion distance		
					Range	Median	Mean	Range	Median	Mean
All (*n* = 105)		66	63	100	0.02–41.1	0.5	19	0–23	6	7
BEN	Fibroadenoma (*n* = 22)	18	81	100	0.1–14.7	0.7	2.5	1–13	6	5
	Cyst (*n* = 32)	0	0	100	0.02–41.1	0.5	1.9	0–16	4	6
	Node (*n* = 5)	2	40	100	0.2–8.9	0.8	2.2	2–23	10	11
	Papillomas (*n* = 4)	4	100	100	0.2–1.0	0.6	0.6	6–20	7	10
	Atheromas (*n* = 2)	2	100	100	0.03–0.6	0.3	0.3	0–1	7	10
MAL	DCIS (*n* = 3)	3	100	100	0.2–3.2	0.6	13	10–20	11	14
	Mamma carcinomas (*n* = 37)	37	100	100	0.05–41.1	0.8	25	0–15	6	6

**Fig. 3:**
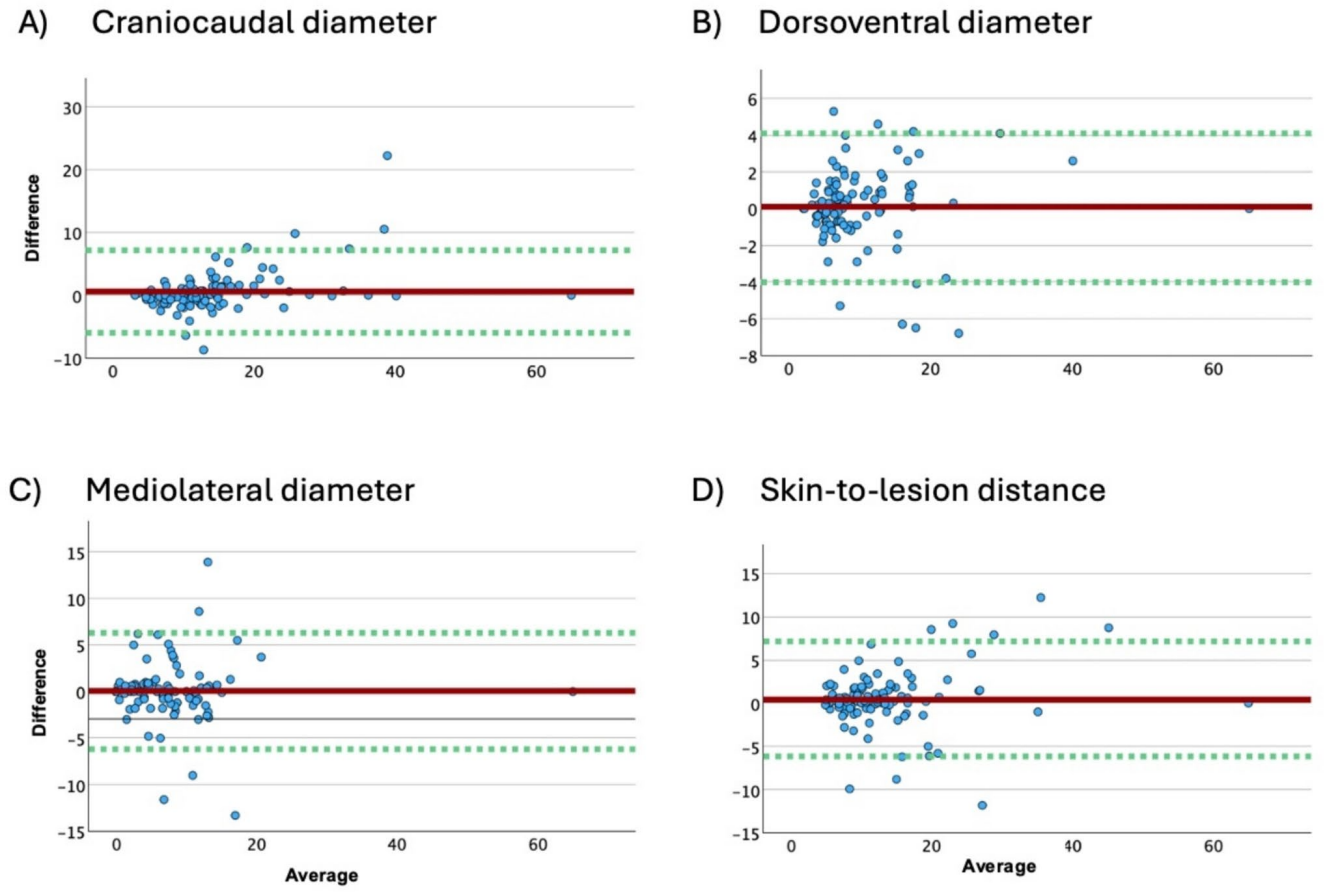
Bland–Altman plot of the evaluated measurements with the stationary high-end ultrasound device (SHUD) and handheld ultrasound device (HHUD). Upper and lower limits of 95%-agreement depicted by the dotted green line and the mean difference depicted by the solid red line. The average and difference are stated in mm

All measured diameters showed an excellent agreement (Table 3). The ICC for craniocaudal diameter, for dorsoventral diameter and mediolateral diameter was 0.936, 0.969 and 0.926, respectively. The ICC for the skin-to-lesion distance was 0.914. The Bland–Altman plots showed no specific pattern so that systematic biases could be ruled out. (Fig. 3). The average differences of all parameters were under 6 mm and the limits of agreement were between − 6.2 mm and 7.2 mm. (Table 3). The results of the ICC and Bland–Altman plots proved that the examination using HHUD and SHUD was highly reliable and not only highly correlated as the high PCC indicated (supp. Table 1).

**Table 3:** Average difference (AD), 95%-limits of agreement (LoA), intraclass correlation coefficient (ICC) and Pearson correlation coefficient (PCC) of the examination results using a stationary high-end ultrasound device (SHUD) and a handheld ultrasound device (HHUD) for the 105 lesions (*n*), presented with 95% confidence interval (CI). Subgroup analysis for benign and malignant lesions as well as fibroadenomas and cysts as major groups of the benign lesions in regards to Intraclass Correlation Coefficient (ICC). The unit for AD and LoA is mm

		Craniocaudal	Dorsoventral	Mediolateral	Skin-to-lesion distance
AD	All (*n* = 105)	0.6	0.1	0.5	0.1
LoA		− 6–7.2	− 4.0–4.1	− 6.1–7.1	− 6.2–6.3
ICC (95% CI)	All (*n* = 105)	0.936 (0.907–0.956)	0.969 (0.954–0.979)	0.926 (0.893–0.949)	0.914 (0.875–0.941)
ICC (95% CI)	Benign (*n* = 65)	0.893 (0.830–0.933)	0.958 (0.932–0.974)	0.884 (0.817–0.928)	0.791 (0.679–0.867)
	Fibroadenoma (*n* = 22)	0.865 (0.703–0.942)	0.954 (0.892–0.981)	0.830 (0.635–0.926)	0.958 (0.901–0.982)
	Cyst (*n* = 32	0.929 (0.859–0.965)	0.944 (0.889–0.972)	0.872 (0.754–0.935)	0.852 (0.837–0.961)
	Malignant (*n* = 40)	0.978 (0.958–0.988)	0.968 (0.940–0.983)	0.950 (0.908–0.973)	0.984 (0.941–0.983)

The subgroup analysis “lesions’ type” revealed an excellent agreement in the case of the measurement of malignant lesions using SHUD and HHUD. Benign lesions examined by SHUD and HHUD had only a good agreement regarding the craniocaudal diameter, the mediolateral diameter, and the skin-to-lesion distance, whereas excellent agreement was seen in the measurement of the dorsoventral diameter. The subdivision of the benign lesions revealed that the measurements of the craniocaudal and the mediolateral diameters of fibroadenomas had a good agreement instead of the excellent agreement seen in the craniocaudal and mediolateral measurement of cysts. The agreements of the dorsoventral diameters were independent from the type of benign lesions. The agreement of the skin-to-lesion distance was excellent in the examination of a fibroadenoma and only good when examining a cyst. The lowest ICC was seen in the mediolateral diameter of fibroadenomas but were still good (Table 3). Due to the similar values of the ICC and PCC, a systematic bias is unlikely (supp. Table1).

When considering the subgroup analysis “skin-to-lesion distance”, in skin-to-lesion distances < 5 mm, the examination using SHUD and HHUD showed an excellent agreement in the measurement of the dorsoventral diameter, a good agreement in the measurement of the craniocaudal and mediolateral diameters, and a moderate agreement in the measurement of the skin-to-lesion distance, while in skin-to-lesion distances ≥ 5 mm, there was an excellent agreement when measuring the craniocaudal, dorsoventral, and mediolateral diameters and a good agreement when measuring the skin-to-lesion distance (Table 4). Based on the PCC and ICC, a systematic bias can be excluded for all parameters (supp. Table1).

**Table 4:** Intraclass correlation coefficient (ICC) of the examination results using a stationary high-end ultrasound device (SHUD) and a handheld ultrasound device (HHUD) for 105 lesions (*n*) analyzed regarding the skin-to-lesion distance and the lesions’ volume as an influencing factor, presented with 95% confidence interval (CI)

ICC (CI 95%)	Craniocaudal diameter	Dorsoventral diameter	Mediolateral diameter	Skin-to-lesion distance
All (*n* = 105)	0.936 (0.907–0.956)	0.969 (0.954–0.979)	0.926 (0.893–0.949)	0.914 (0.875–0.941)
Skin-to-lesion distance < 5 mm (*n* = 44)	0.848 (0.738–0.914)	0.928 (0.871–0.960)	0.833 (0.713–0.905)	0.547 (0.299–0.726)
Skin-to-lesion distance ≥ 5 mm (*n* = 61)	0.983 (0.971–0.990)	0.978 (0.963–0.987)	0.962 (0.967–0.988)	0.899 (0.937–0.977)
Volume < 0.5 ml (*n* = 52)	0.803 (0.682–0.882)	0.719 (0.558–0.828)	0.702 (0.534–0.817)	0.816 (0.699–0.891)
Volume ≥ 0.5 ml (*n* = 53)	0.862 (0.772–0.918)	0.944 (0.905–0.967)	0.853 (0.758–0.913)	0.804 (0.680–0.883)

For the subgroup analysis “lesions’ size”, cases were grouped into lesion volumes ≥ 0.5 ml (*n* = 53) or < 0.5 ml (*n* = 52). For all parameters except for the skin-to-lesion-distance and the craniocaudal diameter, the agreements were higher in cases with a volume ≥ 0.5 ml in comparison to cases with a volume < 0.5 ml (Table 4). In the case of a volume ≥ 0.5 ml, the measurements of a dorsoventral diameter had an excellent agreement between HHUD and SHUD, whereas the measurement of the craniocaudal and mediolateral diameters had a good agreement. In comparison to the lesions’ volume ≥ 0.5 ml, the agreement between the dorsoventral and mediolateral diameter was moderate when examining volumes < 0.5 ml. The agreement between the craniocaudal diameter and the skin-to-lesion distance was good independent of the subgroup. Based on the similar values of the PCC and ICC, a systematic bias can be excluded for all parameters (supp. Table 1).

## Discussion

The sonographic impression classified using the BI-RADS criteria during the examination with HHUD was fully in agreement with those determined using a SHUD (Tab.2). All available histology results demonstrated exact agreement between ultrasound-based dignity evaluations and histological results regardless of the device. In 39 cases, no histological analyis was indicated based on the sonographic impression and BI-RADS categorization [3, 28, 36] so that the diagnoses based on the ultrasound devices in these cases lack a histological verification. Due to the focus on ultrasound devices and due to the existing validation of BI-RADS criteria and lesions type [3], biopsies of all 39 lesions categorized as BI-RADS 2 and 3 would unnecessarily harm participants. The results of this pilot study demonstrate, so far, that HHUD operated by qualified examiners offer equal diagnostic value in comparison to SHUD.

The measurements examined by HHUD and SHUD showed excellent agreement and high correlations for all the measured sonographic parameters and thus are in line with study findings in other fields like obstetrics [17–22]. The Bland–Altman plots suit these PCC and ICC results. The limits of agreement are in an acceptable range for the intended use, whereby results close to the defined thresholds may lead to different treatments: for example, the application of Pembrolizumab for triple-negative breast cancer is often decided based on lesions’ size over or under 20 mm [37, 38]. In comparison to the known studies in gynecology, this study analyzes the reliability in a context of high-frequency ultrasound waves as used for breast examination. So far, only a case report comparing the examination of a non-palpable anti-contraceptive implant using HHUD and SHUD [17] analyzed the application of HHUD using high frequencies during a gynecologic examination. This case report and this study agree on the good applicability and comparable results between SHUD and HHUD in a high-frequency setting.

The subgroup analysis allows a more detailed view regarding the application of the HHUD: The different ICC and PCC regarding the type of lesions could be due to a more thorough and careful examination by the physician when the scanned lesions appear to be cancerous and the clinical consequence would be more serious [39]. On the other hand, the shape of the lesions could impact the ICC and PCC: fibroadenomas are horizontal [36], ovoid, smooth, and solid lesions [40] so that the craniocaudal and mediolateral diameter can change erroneously. In the case of a little deviation in the craniocaudal and mediolateral diameter, the diameter of the fibroadenoma can change due to its oval shape in every examination and thus explain the presented ICC and PCC. In contrast, the dorsoventral diameter of a horizontal fibroadenoma with an oval shape may be more similar at different sonographic cross sections and thus may explain the excellent agreement in this parameter. Cysts are often round lesions [41, 42] and thus lack the dependency of the sonographic cross section and the diameter. Irregular carcinomas may present certain hallmarks so that the examiners are able to better orientate themselves and thus measure similar diameters despite their unknown value of the previous examination with the other device. Therefore, the slightly different ICCs and PCCs in the subgroup analysis “lesions’ type” could be explained by the lesions’ shape.

The lower ICC and PCC by a skin-to-lesion distance under 5 mm in comparison to a skin-to-lesion distance over 5 mm could be explained by a similar phenomenon as seen by the examination of the postpartal uterus [41, 42]: a too small acoustic window for the short distance between transducer and lesion, especially when considering large lesions, could prevent a complete portrayal of the lesion.

Regarding the subgroup analysis “lesions’ volume”, the moderate agreement and the correlation between the dorsoventral and mediolateral diameter in the case of small volumes may be caused by the measurement of the lesion using fingers on a small screen when using the HHUD instead of using the computer mouse when applying the SHUD. This low agreement rate lacks support from other studies, where small distances such as the uterine cavum were measured as well [21]. A distortion due to the calculation of the volume independent of the lesions’ shape may influence these results. This approach was chosen to analyze every lesion similarly and thus independent of their type, which correlates with specific shapes. Analysis combining both parameters, volume and skin-to-lesion distance, could prove the dependence between acoustic window and lesions’ volume but would require a highly powered study.

Regardless of subgroup analysis, the mediolateral diameter seems to be measured most variably when using both devices as it provides the lowest ICC and PCC in comparison to the other diameters. This may be caused by the downward inclination of the ribs. However, even the lowest agreement and correlation, only revealed in specific situations such as in lesions with a skin-to-lesion distance under 5 mm or with a volume under 5 mm, was still moderate. In most cases, agreement and correlation between HHUD and SHUD were excellent and thus argue for the HHUD’s use in breast health, especially in specific situations, where mobility is required.

Several limitations must be acknowledged: 105 lesions from 84 participants were included in this study representing cross sections of breast patients in North Rhine-Westphalia, Germany. To generalize these results, further studies, particularly in other countries, would be necessary. The fact that the examinations were performed by trained professionals influences the agreement and correlation coefficients, so that the results could differ if the examiners lacked ultrasound training. The same examiner performed the ultrasound examinations sequentially with both devices. Thus, the examiners could be influenced by the knowledge of lesion morphology and measurements obtained with the first device on the description, categorization, and measurement during the examination with the second device. The order of the used ultrasound devices was randomly assigned to reduce this influence on the analysis. Another study design, where two examiners with different devices ensured blinding, would lead to an inter-rater bias. In several studies, inter-rate agreement is lower than the intra-rate agreement [43, 44] so that this bias would complicate the evaluation of the reliability of HHUD and SHUD. The fact that only 84 participants with 105 lesions were included, leads to overlapping participants and thus clustered data. Every lesion was individually examined and measured. Due to the focus on the examination’s methods, only lesion-related factors were analyzed. Demographic factors were not included in statistical analysis but may impact interpretation: the inclusion of multiple lesions from one participant leads to overlapping features of the participants, such as skin thickness and breast density, which may influence the results, so that further studies are necessary. The study’s participant selection criteria lead to an imbalance between benign and malignant lesion findings: breast lesions of malignant origin represent only 3–6% of total breast lesions [45]. The cancerous lesions in our study totaled 40 out of 105 (38%), thus potentially limiting the study’s representativeness about lesions’ type but suits our research aim to analyze the HHUD reliability when examining cancerous breast lesions. Different patient motivations to participate in clinical studies could explain these findings. Further imbalances were detected regarding the demographic factors such as age or body mass which led to unbalanced subgroups and may influence statistical analysis. The criteria for the subgroup of lesions’ volume and of the skin-to-lesion distance close to the median may suit the number of participants, but a high-powered study would allow an analysis with other cut-off values and may lead to different results. However, these results imply an influence of these factors. The bra size could be a factor for another subgroup analysis as analyzed in several other breast-related studies [46, 47]. This study used only one specific portable ultrasound device based on silicon chips, whereby almost every ultrasound manufacturer now distributes their one portable ultrasound device based on piezoelectric technology. These devices have their own technical specifications and platforms, so several comparisons between these devices based on study-specific questions have been published [13, 14]. Thereby, this study focuses on the reliability of new ultrasound technology in comparison to the SHUD, so a well-known, established device as a comparison model was used instead of more recently commercialized portable ultrasound devices based on piezoelectric technology. The participants’ and examiners’ opinion were not explored in this study. Several studies concentrated on the positive and negative feature of HHUD [13, 14, 17, 20–22]. Such considerations would need standardized questionnaires and, in case of evaluation of examiners’ opinion, more examiners.

## Conclusion

Portable handheld ultrasound devices based on silicon chips demonstrate high reliability and clinical potential as alternatives for stationary ultrasound systems in the evaluation of breast lesions. The portability and cost-effectiveness of HHUD combined with their demonstrated reliability make them suitable for expanding high-quality breast imaging services to locations without conventional ultrasound access. Thus, POCUS in breast health could serve as a flexible, powerful diagnostic standard which functions effectively in pre-, intra-, and postoperative settings as well as in rural outreach programs, emergency departments and specialized facilities. 

## Supplementary Information

Below is the link to the electronic supplementary material.

Supplementary file1 (DOCX 14 KB)

## Data Availability

No datasets were generated or analyzed during the current study.
