# Supplementary material for: Breast assessment using next generation handheld ultrasound device based on silicon chips: a pilot study in senology
Source: Arch Gynecol Obstet. 2026 Jan 24;313(1):61. doi: 10.1007/s00404-026-08326-1 (PMC12831706; doi:10.1007/s00404-026-08326-1)
Supplement: Supplementary file 1 — Supplementary file1 (DOCX 14 KB) [file 404_2026_8326_MOESM1_ESM.docx]

Suppl. Table 1 Pearson correlation coefficient (PCC) of the examination results using a stationary high-end ultrasound device (SHUD) and a handheld ultrasound device (HHUD) for 105 lesions (n: number). Subgroup analyses were performed for benign and malignant lesions as well as fibroadenomas and cysts as major groups of the benign lesions, for skin-to-lesion distance and for lesions’ volume as an influencing factor.

| PCC  Subgroup | Craniocaudal diameter | Dorsoventral diameter | Mediolateral diameter | Skin-to-lesion distance |
| --- | --- | --- | --- | --- |
| All  (n=105) | 0.946 | 0.969 | 0.928 | 0.914 |
| Benign (n=65) | 0.925 | 0.967 | 0.898 | 0.795 |
| *Fibroadenoma*  *(n=22)* | *0.908* | *0.955* | *0.841* | *0.958* |
| *Cyst (n=32)* | *0.954* | *0.962* | *0.906* | *0.852* |
| Malignant  (n=40) | 0.979 | 0.968 | 0.950 | 0.968 |
| Skin-to-lesion distance <5mm (n=44) | 0.910 | 0.929 | 0.854 | 0.600 |
| Skin-to-lesion distance ≥5mm (n=61) | 0.984 | 0.978 | 0.962 | 0.899 |
| Volume <0.5ml (n=52) | 0.820 | 0.725 | 0.715 | 0.816 |
| Volume ≥0.5ml (n=53) | 0.905 | 0.944 | 0.869 | 0.804 |
